# Genetic Characteristics Associated With Drug Resistance in Lung Cancer and Colorectal Cancer Using Whole Exome Sequencing of Cell-Free DNA

**DOI:** 10.3389/fonc.2022.843561

**Published:** 2022-03-24

**Authors:** Jong Won Lee, Young Soo Park, Jung Yoon Choi, Won Jin Chang, Soohyeon Lee, Jae Sook Sung, Boyeon Kim, Saet Byeol Lee, Sung Yong Lee, Jungmin Choi, Yeul Hong Kim

**Affiliations:** ^1^ Cancer Research Institute, Korea University College of Medicine, Seoul, South Korea; ^2^ Brain Korea 21 Plus Project for Biomedical Science, Korea University College of Medicine, Seoul, South Korea; ^3^ Department of Biomedical Sciences, Korea University College of Medicine, Seoul, South Korea; ^4^ Division of Hematology–Oncology, Department of Internal Medicine, Korea University Ansan Hospital, Korea University College of Medicine, Gyeonggi-do, South Korea; ^5^ Division of Hematology–Oncology, Department of Internal Medicine, Korea University Anam Hospital, Korea University College of Medicine, Seoul, South Korea; ^6^ Division of Pulmonary, Allergy and Critical Care Medicine, Department of Internal Medicine, Korea University Medical Center, Korea University College of Medicine, Seoul, South Korea; ^7^ Department of Genetics, Yale University School of Medicine, New Haven, CT, United States

**Keywords:** circulating cell-free DNA (cfDNA), whole exome sequencing, drug resistance, lung cancer, colorectal cancer

## Abstract

Circulating cell-free DNA (cfDNA) can be used to characterize tumor genomes through next-generation sequencing (NGS)-based approaches. We aim to identify novel genetic alterations associated with drug resistance in lung cancer and colorectal cancer patients who were treated with EGFR-targeted therapy and cytotoxic chemotherapy through whole exome sequencing (WES) of cfDNA. A cohort of 18 lung cancer patients was treated with EGFR TKI or cytotoxic chemotherapy, and a cohort of 37 colorectal cancer patients was treated with EGFR monoclonal antibody or cytotoxic chemotherapy alone. Serum samples were drawn before and after development of drug resistance, and the genetic mutational profile was analyzed with WES data. For 110 paired cfDNA and matched germline DNA WES samples, mean coverage of 138x (range, 52–208.4x) and 47x (range, 30.5–125.1x) was achieved, respectively. After excluding synonymous variants, mutants identified in more than two patients at the time of acquired resistance were selected. Seven genes in lung cancer and 16 genes in colorectal cancer were found, namely, APC, TP53, KRAS, SMAD4, and EGFR. In addition, the GPR155 I357S mutation in lung cancer and ADAMTS20 S1597P and TTN R7415H mutations in colorectal cancer were frequently detected at the time of acquired resistance, indicating that these mutations have an important function in acquired resistance to chemotherapy. Our data suggest that novel genetic variants associated with drug resistance can be identified using cfDNA WES. Further validation is necessary, but these candidate genes are promising therapeutic targets for overcoming drug resistance in lung cancer and colorectal cancer.

## Introduction

Significant progress has been made for tracking tumor mutations in cell-free DNA (cfDNA) in the last decade (1–3). cfDNA is thought to be released into circulation by necrotic or apoptotic cells, and is frequently present at higher quantities in patients with cancer than in healthy individuals (4). An analysis of those cfDNA can be used as a surrogate marker for molecular diagnosis, and for surveillance of tumor progression (5). These techniques enable an access of tumor molecular information when tumor biopsies are intractable, particularly for patients with metastatic cancer.

Whole exome sequencing (WES) of cfDNA has demonstrated potential to detect clinically relevant alterations (6). Although significant progress has been made for tracking previously detected tumor mutations using targeted gene panels or single gene assays, WES enables a more comprehensive analysis covering the complex landscape of somatic alterations (7). Also, WES analyses of cfDNA hold great promise to identify emerging genetic alterations that are of interest in treatment of drug resistance.

Lung cancer and colorectal cancers are the two leading cancer causing mortalities in both men and women in Korea during 2021 (8). Lung cancer and metastatic colorectal cancer (mCRC) are often diagnosed at an advanced stage when tumor cell dissemination has taken place (9). The treatment of the RAS wild-type mCRC is currently based on the use of chemotherapy doublets (fluoropyrimidine and oxaliplatin or irinotecan) and biological drug (cetuximab, bevacizumab, panitumumab) (10). This concept is well expressed in the ESMO (European School of Medical Oncology) guidelines (11). In lung cancer, the use of EGFR tyrosine kinase inhibitors (EGFR-TKIs) is now a common practice for the first-line treatment of patients with EGFR sensitizing mutation, leading to longer progression-free survival (PFS) intervals with fewer or at least different side effects than chemotherapy (12). Mechanisms of acquired resistance to targeted therapy in both types of cancer have been largely deciphered over the past 20 years and targeting those genetic driver changes are already in clinical use or under clinical investigation (13). Despite great promises brought by the new paradigm of targeted therapy, the invariable emergence of acquired drug resistance not only limits the duration of tumor response but also represents the major obstacle for more meaningful impact on long-term survival in genotype-matched precision medicine (14). The study of sequential liquid biopsies, obtained at baseline and at the moment of progression, from lung cancer and mCRC patients has allowed the identification of new genetic alterations, which explain the development of acquired resistance.

There are a few reports of attempts to analyze cfDNA WES data as a platform for non-invasive analysis of tumor evolution during cancer treatment (4, 5, 15). Yet, the study with a large number of patients in lung cancer and colorectal cancer was never investigated. Our purpose in this investigation was to perform WES of serum cfDNA in patients with lung cancer and colorectal cancer. From the analysis of those data, novel genetic variants associated with drug resistance could be identified.

## Materials and Methods

### Patients and Sample Selection

Patients with metastatic colorectal cancer (mCRC) and advanced lung cancer who had been treated with adjuvant chemotherapy at the Korea University Anam Hospital and Guro Hospital were reviewed. From January 2010 to December 2019, patients were treated with chemotherapy (Lung cancer—EGFR TKI or Cytotoxic chemotherapy), (Colorectal cancer—Cytotoxic chemotherapy or Cetuximab) and total of 42 lung cancer patients and 63 colorectal cancer patient samples were used for this study if serum samples for WES of the baseline and at acquired resistance were both available. Both blood samples from baseline and resistance time points were used for cfDNA extraction and WES. Tumor response was determined in accordance with the Response Evaluation Criteria in Solid Tumors 1.1 (RECIST 1.1) guidelines (16). Tumor size was measured using summation of the longest diameter of two largest tumors. If a lesion is smaller than 5 mm, it was recorded as non-measurable. The study was approved by the institutional review boards of the Korea University Anam Hospital and Guro Hospital, and informed consent was obtained.

### Blood (Serum) Sampling

Blood samples were obtained at diagnosis and subsequently in several-month intervals during treatment and follow-ups. Blood samples were collected in SST Vacutainer tubes (Yellow top) for serum isolation. For serum isolation, tubes were centrifuged at 2,500 rpm for 7 min at RT, and the supernatant fraction transferred to a fresh tube and re-centrifuged at 16,000×*g* for 10 min at 4°C. The supernatant fraction from the second centrifugation was transferred to a cryotube for storage in a −80°C freezer in our laboratory within 1 to 4 h after collection.

### cfDNA and Germline DNA Extraction

cfDNA was extracted from 1 to 2 ml of serum using a Qiagen circulating nucleic acid kit (Qiagen,Germany) with the Quavac24s system, according to the recommendation of the manufacturers. When required, additional purification was performed using Agencourt AMPure XP (BeckMan Coulter, Brea, CA) to remove larger contaminating nucleic acid. cfDNA concentration and quality were measured by Tapestation or Agilent 2100 Bioanalyzer (Agilent, Santa Clara, CA) using the High sensitivity DNA kit. Germline DNA was extracted from PBMCs using Qiagen blood minikit (Qiagen) according to the instructions of the manufacturer.

### Library Preparation and WES

For cfDNA library preparation, an average 10 ng of cfDNA were engaged without an initial fragmentation and Agilent SureSelect Human All Exome V4 Kit and Twist Human Core Exome Kit were used according to instructions of the manufacturer. WES was performed on serum samples from 105 patients using an Illumina HiSeq 2500, with 100-bp paired-end reads.

### Bioinformatic Analysis (Pipeline)

Sequence QC was done through FastqQC 0.11.2 (17), and it was mapped to human reference genome sequence NCBI b37 using bwa 0.7.12 (18). BAM files were realigned with the Genome Analysis Toolkit 4.1 (19) (GATK) IndelRealigner, and base quality scores were recalibrated by the GATK base quality recalibration tool. WES variants calling was performed using two variant callers with 1% cut off value: GATK’s Mutect2 v4.1.4.1 (20) return only SNVs and Strelka2 (21) returns the lowest number of both SNV and indel calls according to their somatic pipeline, respectively. Final variants were annotated using ANNOVAR-v2021-06-07 with build hg19 databases, namely, refGene, dbNSFP version 2.6, COSMIC database version 70, NHLBI-ESP project with 6,500 exomes, 1000 Genomes Project, dbSNP 138, CLINVAR database, Polypen2, COSMIC, ICGC and functional prediction was performed. SNVs with quality <30, a depth of coverage <20 in cfDNA samples, or <3 reads supporting the variant were filtered out. Only within exons of coding genes or splicing sites were kept. Then, variants reported in more than 1% of the population in the 1,000 genomes or Panel of Normal of Exome Sequencing of Korean population (22) were discarded to filter out polymorphisms. Finally, synonymous variants were filtered out except those with a COSMIC ID. Subsequently, all identified somatic mutations were manually examined by visual inspection of the BAM files to remove false positive calls as were located in repetitive areas and variants with many adjacent variants as they were suspected to result from systemic misalignment.

Copy number variation analysis was performed using FACETS V0.5.6: https://github.com/mskcc/facets (23). ctDNA fraction was estimated by FACETS from data of the WES cfDNA sample and absolute copy number (ABCN) were called depends on tumor fraction estimation from cfDNA as previously described (24), and mean tcn.em values were used. To estimate ctDNA amount, mean tcn.em values were used to calculate ctDNA content of total cfDNA (25).

Mutational signature analysis was performed using the deconstructSigs package in R (26), Signal (27) or MuSiCa (28) that selects which combination of known mutational signatures can account for the observed mutational profile in each sample as previously described (29).

### ddPCR

Mutant allele frequency was assessed using the QX200 Droplet Digital PCR (ddPCR) System (BioRad, Milan, Italy) in accordance with the instructions of the manufacturer. The PrimePCR™ ddPCR™ Mutation Assay (BioRad) for humans was used. This kit evaluates KRAS p.G12C and KRAS WT for p.G12C, KRAS p.G12R and KRAS WT for p.G12R, KRAS p.G12V and KRAS WT for p.G12V, KRAS p.G12D and KRAS WT for p.G12D, KRAS p.G12S and KRAS WT for p.G12S, KRAS p.G13D and KRAS WT for p.G13D, KRAS p.G13C and KRAS WT for p.G13C, NRAS p.Q61R and NRAS WT for p.Q61R, EGFR p.E746_A750del and EGFR WT for p.E746_A750del, EGFR p.L858R and EGFR WT for p.L858R, and BRAF p.V600E and BRAF WT for p.V600E. ddPCR reaction mixtures contained a final concentration of 250 nM of each of the probes, 900 nM of forward and reverse primers, 1× ddPCR Supermix for Probes (Bio-Rad), and 0.7–3 ng cfDNA in a final volume of 20 μl. Each reaction included a blank sample corresponding to H2O, another corresponding to wild-type DNA, and a positive control (KRAS p.G12D, EGFR p.E746_A750del and EGFR p.L858R) using HD780 Reference Standard Set (Horizon, Cambridge, UK). The steps are described in more detail as previously (30).

### Statistical Analysis

Statistical analysis was performed using R software (version 4.0.3). Pearson’s correlation coefficient R >0.5 was considered to indicate a strong correlation. Survival curved were plotted using the cBioPortal and Kaplan–Meier plots as previously described (31). All results are displayed with P-values from a log-rank test. A P-values of <0.05 were considered to be statistically significant.

## Results

### Overall Study Design and Patient Characteristics

The study comprised a major aim to identify the somatic variants associated with drug resistance to chemotherapy in lung cancer and colorectal cancer using circulating cell free DNA. For this purpose, we performed the whole exome sequencing of serum samples from 55 patients with stage III or IV cancer (18 lung cancer and 37 colorectal cancer patients) which were drawn before and after the development of drug resistance. Patient characteristics, namely, clinical and histological features in this study are detailed in **Table 1**. The mean age was 65 and 64 years old in lung cancer and colorectal cancer, respectively. In lung cancer, all tumor types being treated were non-small cell lung cancer and the patients received standard cytotoxic chemotherapy (38.9%) or EGFR-TKI (61.1%). In colorectal cancer, all the tumor types being treated were adenocarcinoma and all patients were treated with a modified standard cytotoxic chemotherapy (78.4%) or additional EGFR monoclonal antibody cetuximab (21.6%). With this approach, we could expect to find not only drug specific genetic variants, but also common variants regardless of drug type.

**Table 1:** Patient characteristics.

	Lung cancer (n = 18)		Colorectal cancer (n = 37)	
	Cytotoxic chemotherapy	EGFR-TKI	Cytotoxic chemotherapy only	Chemotherapy with Cetuximab
	N = 7	N = 11	N = 29	N = 8
**Sex**				
** Female**	**3**	**4**	**12**	**2**
** Male**	**4**	**7**	**17**	**6**
**Age at diagnosis (mean years ±SD)**	**62 ± 12**	**67 ± 7**	**65 ± 14**	**60 ± 9**
**Histology (Lung cancer)**				
** NSCLC**	**5**	**11**		
** Adenocarcinoma**	**1**			
** Squamous**	**1**			
** SCLC**				
**Histology (Colorectal cancer)**				
** Adenocarcinoma**			**29**	**8**

### cfDNA WES Analysis and Bioinformatics Pipeline

**Figure 1:**
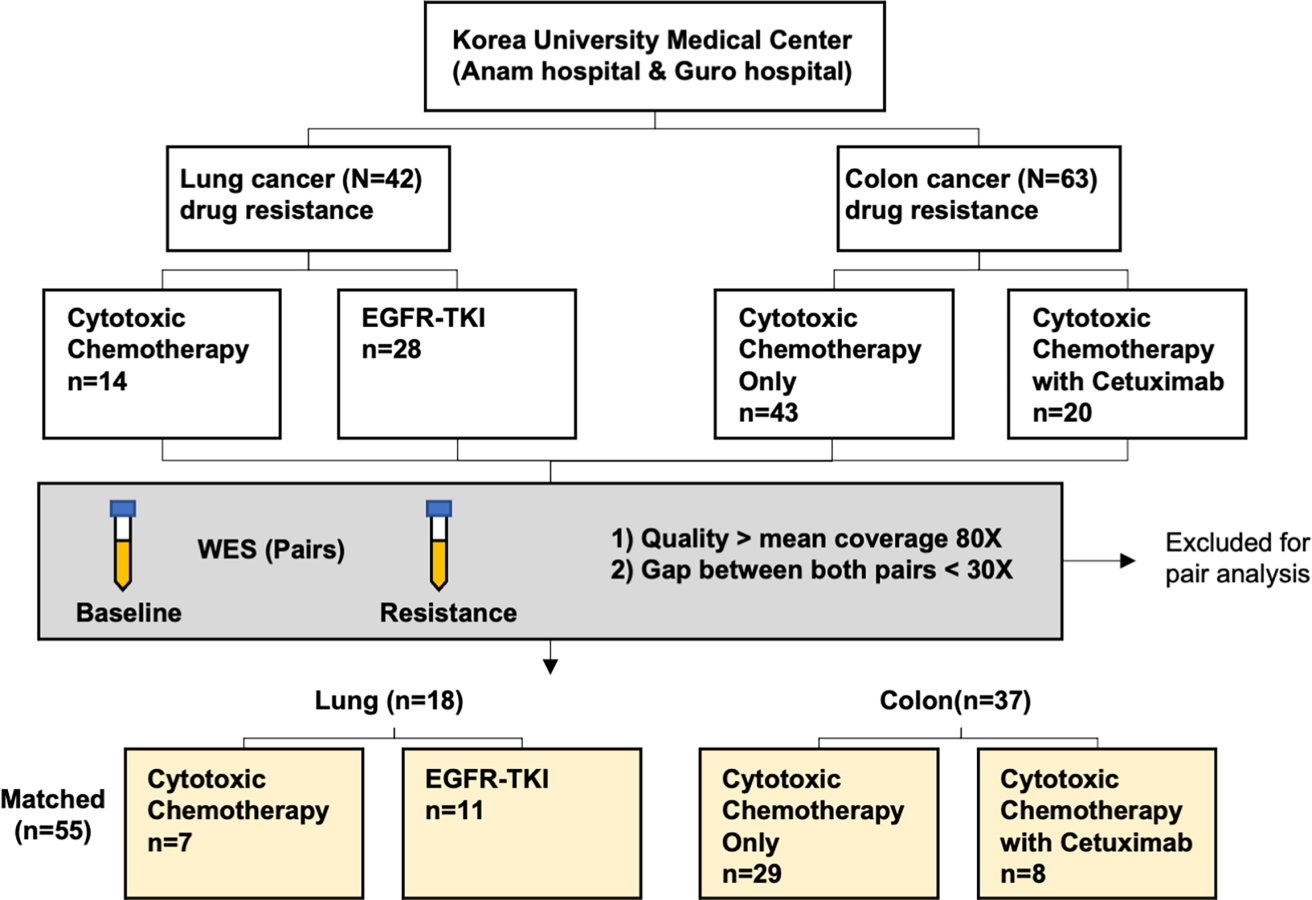
Patient recruitment and enrollment flow chart.

A total of 210 cfDNA paired samples underwent WES, and it generated for a median 83.5x coverage (range, 20.1–211.5x). For samples over 70x coverages, there were few PCR duplicates, so re-sequencing was performed on those samples that can be expected to increase mean coverage up to 100x. A total of 152 samples (59 in lung cancer + 93 in colorectal cancer) had a median of 91.5x and the rest had a median of 51.8x. Thus, we re-sequenced 152 samples to achieve a median 130.5x (mean = 134.5x) and was used for the downstream analysis. Pair analysis was performed with samples with WES at mean coverage of 80x or more at both baseline and at the time of acquired resistance and gap between the two value less than 30x. Finally, this study included 110 paired serum samples from 18 lung cancer and 37 colorectal cancer patients which were drawn before and after the development of drug resistance. For those 110 paired cfDNA and matched germline DNA WES, a mean coverage of 138x (range, 52–208.4x) and 47x (range, 30.5–125.1x) were achieved respectively, and thus enabling a detection of MAF at 1%. Among these patients, the median age was 65 (range, 24–88) years and there were 34 men (61.8%). Patient enrollment and study overview are presented in **Figure 1** and **Table 1**.

**Figure 2:**
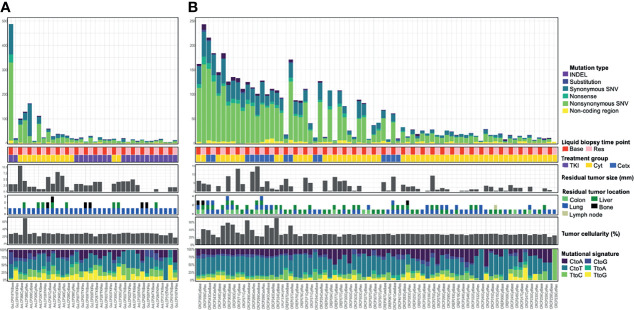
Somatic mutational landscape across lung cancer **(A)** and colorectal cancer **(B)** cfDNA samples analyzed with WES. Top bar graph showed a distribution of somatic mutation count across cfDNA samples in 18 lung cancer patients **(A)** or in 37 colorectal cancer patients **(B)**. The second matrices show liquid biopsy time point or treatment group. Base, baseline; Res, Resistant time point; TKI, EGFR-TKI; Cyt, Cytotoxic chemotherapy; Cetx, Cetuximab. The third bar graphs illustrate residual tumor size, location, and tumor cellularity as a proportion of total cfDNA. The bottom bar graph shows transcriptomic data composition. Samples are ordered by patient and mutation count as determined from WES. Some tiny nodules smaller than 5 mm were non-measurable and not be shown in Residual tumor size section.

For somatic mutation calling from cfDNA WES data, we used two variants callers GATK’s Mutect2 and Strelka2 as described in the *Materials and Methods* section. Circulating Tumor DNA (ctDNA) fraction was estimated by FACETS from the cfDNA WES data and mean cf.em values were used. Analysis based on the FACETS tool revealed a mean 28.2% of ctDNA (range, 17–69.3%) in lung cancer and mean 30.9% of ctDNA (range, 15.7–71%) in colorectal (**Figure 2**). These numbers are comparable to those observed from the cfDNA WES analysis of the other 44 cancer patients (mean 18%, range, 4.5–36.2%) although tumor types are different as metastatic breast and prostate cancer (3). We assessed whether ctDNA content was associated with the number of called somatic mutations or the residual tumor information of the patient. Indeed, we found that the number of somatic variants were associated with ctDNA amount and residual tumor size in colorectal cancer (Pearson’s correlation, rho = 0.25, p-value = 0.033 and rho = 0.27, p-value = 0.02, respectively), but not in lung cancer (**Supplementary Figure S1A**). The total amount of ctDNA did not show significant differences depending on the residual tumor size (**Supplementary Figure S1B**). This result indicates that correlate ctDNA with a number of somatic variants was well reflected in colorectal cancer than lung cancer, and there are similar findings observed in a study of ctDNA with various tumor types (32, 33).

For the 36 serum samples from 18 lung cancer patients, we identified a mean 43.7 SNVs and small indels (range, 5–487). Also, for the 74 serum samples from 37 colorectal cancer patients, a mean 60.5 SNVs and small indels (range, 3–243) were identified. As a result, we identified a total of 1,576 somatic variants in 36 lung cancer serum samples (**Figure 2A**), and 4,480 somatic variants in 74 colorectal cancer serum samples (**Figure 2B**). These numbers are comparable to those observed in one of the largest studies attempted at WES-based TMB quantification from liquid biopsy (mean 140 variants, range, 19–818) which was performed on 32 metastatic patients with various cancer types (34) considering that the amount of input DNA used is half. The samples of the each cohort exhibited outlier highest number of somatic mutation (243–487 SNVs and small indels; 4.8–9.5 Mut/Mb) and can be considered to exhibit a ‘Hypermutator-like condition’ as described in the TCGA study including lung cancer (35). Among an updated inventory of about 276 human DNA repair genes (36), two samples (GuLCP018TKIBase and CRCP343CetxBase) showed more than twelve DNA repair gene variants (**Supplementary Table 1**). Moreover, 23 DNA repair gene variants including BRCA1 were shown in the GuLCP018TKIBase sample.

Next, we examined the composition of six possible base-pair substitutions and found that a high rate of C > T transition, for all groups (**Supplementary Figure S2A**). Consistently on decomposing the mutational spectrum is similar to the trinucleotide signature associated with aging (e.g., COSMIC signature 1) and defective DNA MMR (e.g., COSMIC signature 15), a mutational process that is prevalent in most lung cancer and colorectal cancers (37). The median proportion of signatures 2 and 13 (APOBEC) was higher and signature 24 (Aflatoxin) was lower in baseline group compared with the resistance to EGFR-TKI group in lung cancer.

In a recent study using endometrial cancer, the researchers detected acquired high MSI in ctDNA from one patient whose primary tumor was MSI stable (38). We analyzed the MSI and found that no samples had more than 3% unstable microsatellites (**Supplementary Figure S2B**). These results indicate that those two samples with high number of somatic mutations was potentially explained by DNA repair gene alteration.

### Validation of cfDNA WES Using ddPCR

To validate the dynamic range and accuracy of WES, a subset of samples with cfDNA availability was tested by ddPCR for mutation detection in 4 genes (KRAS, NRAS, EGFR, and BRAF). A total of 14 samples were applied to select variants having at least one read containing the allele of the variants. Orthogonal validation with serum ddPCR for those mutations showed concordant findings to serum WES in 10 of 14 results, and the ddPCR-derived VAFs correlated well with those obtained with WES (Pearson’s correlation = 0.97, P-value = 2.7e−08; **Supplementary Figure S3**). Also, it indicated a high accuracy (12/14 = 85.7%) of ddPCR measurement for those probes with a level of mutant fractional abundance ≥1%. Thus, we applied bioinformatics pipeline that enabled to establish a threshold for SNV detection of 1% by cfDNA WES, below which SNVs were not distinguishable from the background.

### Identification of SNVs Associated With Drug Resistance in cfDNA

**Figure 3:**
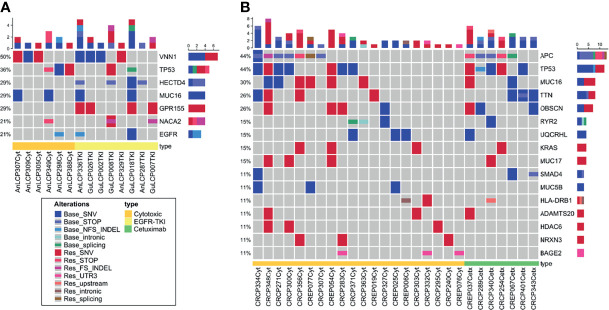
Oncoprint across lung cancer **(A)** and colorectal cancer **(B)** cfDNA samples analyzed with WES. **(A)** Seven frequently mutated genes in 18 lung cancer patients identified through cfDNA WES. **(B)** Sixteen frequently mutated genes in 37 colorectal cancer patients identified through cfDNA WES. This visualization provides an overview of the non-synonymous alterations in particular genes (rows) affecting particular individual patients (columns). Reddish colors indicate increased VAF over 5% or observed at the time point of acquired resistance only (Res_). Bluish colors decreased VAF over 5% or observed at the time of baseline only (Base_).

To evaluated whether the somatic variants in cfDNA related with drug resistance could be identified, the variants at the time of acquired resistance were compared to those in baseline. To identify somatic variants, germline DNA from PBMCs was used as a control. Germline variants and acquired somatic alterations from clonal hematopoiesis are estimated to be removed during this process. After checking the bam files and plot reads and removing false positive, we found a median of 18.5 mutations in lung cancer and 26 mutations in colorectal cancer per patients. After excluding synonymous variants, we selected genes that were changed during observation at the time of baseline only or acquired resistance only. Likewise, increased or decreased VAF over 5% genes with more than two cancer patients were selected. This yielded seven genes in lung cancer, and 16 genes in colorectal cancer, which are plotted in a heat map with one-way hierarchical clustering referring to treatment conditions as shown in **Figure 3**.

Several mechanisms of resistance have been described to anti-EGFR-TKI in lung cancer and anti-EGFR monoclonal antibody in colorectal cancer (39–42). Of those, KRAS, EGFR, and APC were analyzed and KRAS (G12D, G12V), EGFR (Ex19 del, L858R) and APC R213X were detected in seven patients. EGFR Ex19 del and L858R mutations were detected at baseline time point only in three lung cancer patients. Samples with those three patients showed similar tumor cellularity both at baseline and acquired resistance time point and VAF might be not affected by tumor cellularity. This result indicates that clones with those EGFR mutations could be decreased and other resistant clones were expanded.

KRAS G12C, C12V, and G12D variants were detected at resistance time point only or increased in three colorectal cancer patients who were treated with cytotoxic chemotherapy. Various APC nonsense mutations were identified in both baseline and resistance time points. Among them, Hotspot mutation R213X was increased or observed at resistance time point only. These results indicate that previously reported variants related to drug resistance could be identified in cfDNA WES.

### Investigation of Top Frequently Mutated Genes

**Figure 4:**
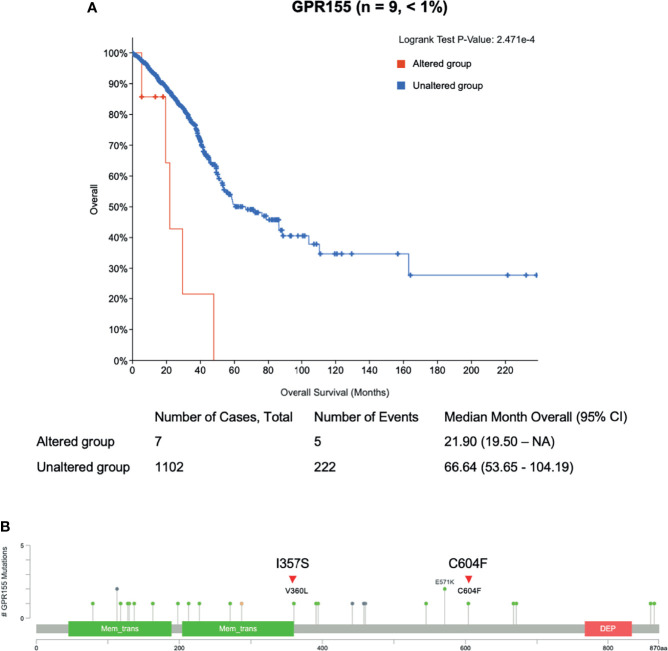
Clinical relevance of GPR155 mutation in lung cancer patients. **(A)** Overall survival analysis of patients with GRP155 alterations (red line) is compared to that of those without alterations (blue line). **(B)** Different mutation sites of GPR155 in lung cancer. Known hotspot mutation sites in COSMIC data are labeled. Each lollipop label shows the amino acid change and its location in the amino acid sequence. Known gene/protein domains are shown in color, and other regions are colored dark gray. Red triangles represent mutations found in this study, I357S (n = 3) and C604F (n = 1).

To evaluate the somatic variants potential for drug resistance, we first focused on the frequently mutated genes in cfDNA with acquired resistant time point. In lung cancer, TP53 gene harbored three mutations, NACA2 and GPR155 genes had two mutations, and VNN1 gene possessed one mutation (**Supplementary Table 2**). Genetic alteration of these genes was visualized as an oncoprint representing missense mutations, nonsense mutations, and non-frameshift substitution (**Figure 3**). Lung cancer patient data and the cBioPortal online tool were used for examine these mutated genes. Among them, GPR155 I357S mutations were estimated as pathogenic (score 0.99) in the COSMIC database, which were not reported in ClinVar. Interestingly, patients with GPR155 alteration showed short overall survival compared those with unaltered patients (**Figure 4A**). In addition, we found that three of four mutations of GPR155 were located in the I357S position; whereas, other genes contained mutations at multiple locations. Detailed mutation sites in GPR155 are shown in **Figure 4B**. Also, GPR155 mutations were observed only in the EGFR-TKI treated group. This result suggests the possibility that GPR155 I357S mutation may contribute to the drug resistance in lung cancer patients especially EGFR-TKI.

In colorectal cancer, TP53, TTN, OBSCN, and MUC17 genes harbored four mutations: MUC16 had five mutations, and HLA-DRB1, ADAMTS20, and HDAC6 genes possessed three mutations. Remaining NRXN3 and BAGE2 genes had two and one mutations respectively (**Supplementary Table 2**). Genetic alteration of these genes was visualized as an oncoprint representing missense mutations, nonsense mutations, non-frameshift substitution, and untranslated region (**Figure 3**). Except well-known cancer related genes TP53, KRAS, and APC, pathogenic variants were estimated in ADAMTS20 S1597P and TTN R7415H only (scores 0.83 and 0.74 respectively). ADAMTS20 was found to be downregulated in colorectal cancer (43) and TTN was reported to be frequently detected in solid tumors including colorectal cancer (44, 45). This result suggests that those gene alterations may lead to the resistance to chemotherapy in colorectal cancer patients.

### Copy Number Variants (CNVs)

**Figure 5:**
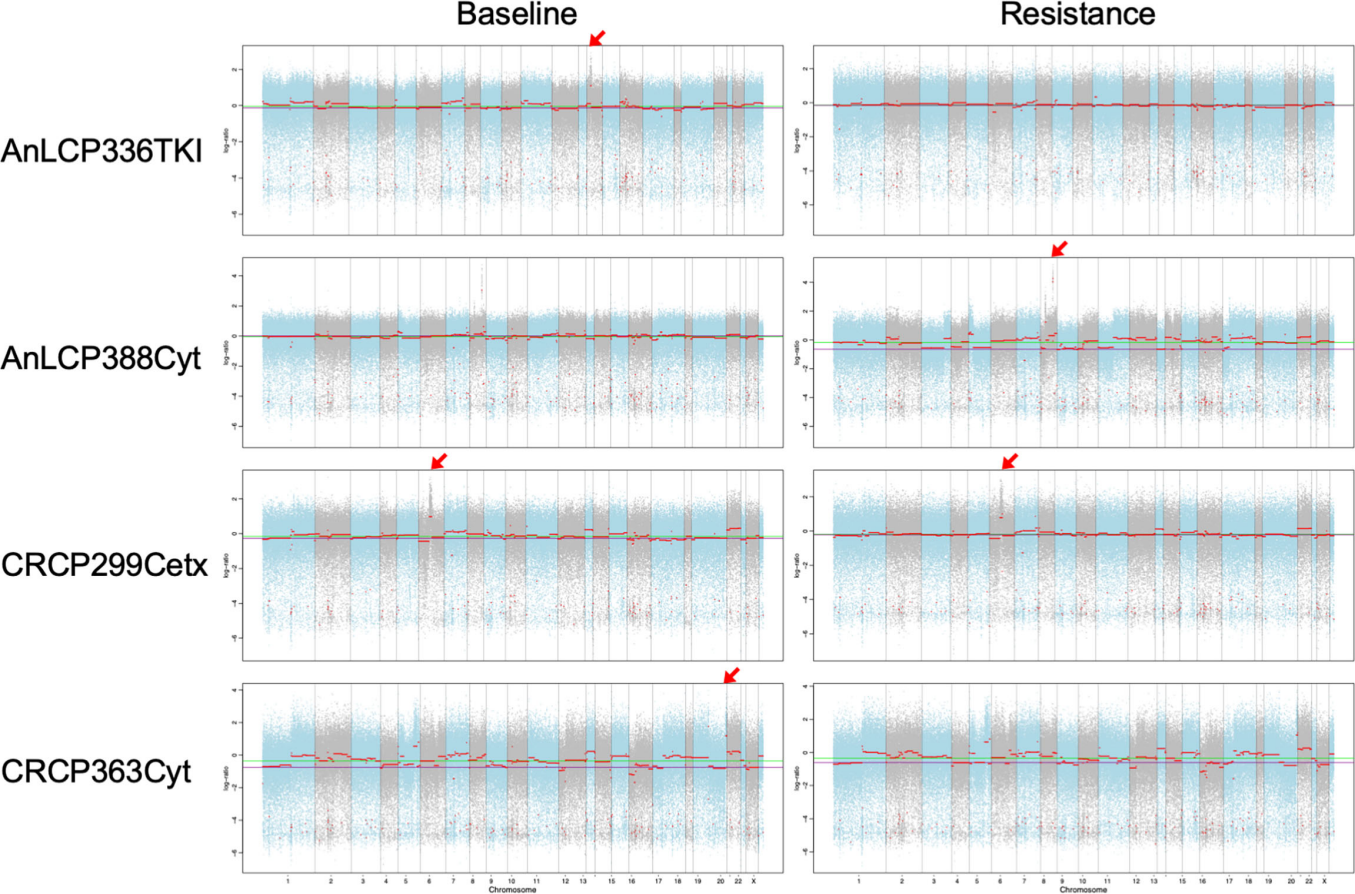
Copy number variants detected in cfDNA using WES from AnLCP336, AnLCP338, CRCP299, and CRCP363. The red arrows indicate the top amplified region including cancer-related genes annotated by Oncomine and Cosmic567.

To evaluates whether the somatic CNVs in cfDNA related with drug resistance could be identified, the variants at the time of acquired resistance were compared to those in baseline. Significantly amplified peaks for those two groups were identified using FACETS as described in the *Materials and Methods* section. To select samples with top amplified regions, mean tcn.em values larger than 8 are categorized as “gain”. Here, we identified CNVs in cfDNA of lung cancer and colon cancer patients with WES and found that gains in chromosomes 1, 6, 7, 8, 10, 14, 16, 19, and 20. Among them, regions including cancer related genes annotated by Oncomine and Cosmic567 were selected (**Figure 5** and **Supplementary Table 3**). In the case of AnLCP388Cyt, we observed focal amplification of the 8q24 and 8p11 chromosomal region in an acquired resistant time point. This region containing MYC and FGFR1 genes, and MYC copy number gains in the patients with primary resistance were reported as higher than in the sensitive patients against EGFR-TKI treatment (46). FGFR1 was frequently amplified in squamous cell lung cancer and this indicates that the mechanism of acquired resistance in this patient might be the activation of pathway through MYC and FGFR1 (47). In case of AnLCP336TKI, focal amplification of the 14q13 chromosomal region was observed only in baseline time point. This region containing NKX2-1 and NKX2-8 genes were reported as prognostic factors in lung cancer (48). In the case of CRPC363Cyt, focal amplification of the 19q12 chromosomal region including CCNE1 was also observed in baseline only. In the case of CRCP299Cetx, focal amplification of the 6p21 chromosomal region including CCND3 was observed in both baseline and acquired resistance time points, which suggests that different resistance mechanism would be involved.

**Table:** 

Review Table
Novel genetic variants associated with drug resistance could be identified through cell-free DNA (cfDNA) whole exome sequencing in lung cancer and colorectal cancer patients.
For cfDNA WES, mean coverage of 138x (range, 52–208.4x) was achieved, and a threshold for SNV detection of 1% was established by ddPCR validation.
GPR155 I357S mutation in lung cancer and ADAMTS20 S1597P and TTN R7415H mutations in colorectal cancer were frequently detected at the time of acquired resistance.
Increased detection indicates that these mutations may have an important function in acquired resistance to chemotherapy.

## Discussion

In this work, we used WES based on cfDNA liquid biopsy for 55 lung and colorectal cancer patients to identify novel somatic variants associated with drug resistance after treatment including cytotoxic chemotherapy or EGFR targeted therapy. Recently, a wide range of genomic alterations have reported association with cancer behavior, and most of the alterations are generally found in coding regions (49). Therefore, WES is a rational strategy for identifying novel somatic variants associated with drug resistance. To identify somatic variants only, alterations that could possibly be regarded to come from a normal cell were excluded during our analysis using matched normal sample and previous SNP databases. Variants resulting from clonal hematopoiesis could be estimated to be removed using the sequencing results from matched leukocytes as a reference.

It is hypothesized that cfDNA is released from tumor cells through various cell physiological events such as apoptosis, necrosis and secretion into the blood circulation (50). Numerous studies have shown that tumor-derived cfDNA better reflects the complete genetic landscape of the tumor compared to tissue biopsies. Apart from also offering the additional benefits of longitudinal sampling, the analysis of cfDNA represents a promising modality for sequential monitoring of the molecular response of cancer during targeted therapy (51). However, cfDNA profiling also has limitations. Although it is possible that some patients did not have alterations in gene covered by the NGS assay, in most cases, the lack of detection of genomic alterations in cfDNA was likely due to other factors, namely, low tumor burden, lack of cfDNA shedding by some tumors, and timing of blood collection (52). The major technical issue with this approach has been assay specificity and sensitivity. A major drawback of cfDNA assay is the low frequency of some of the mutations that occur in tumors. Low sequencing coverage used for WES resulting in false-negative results for cfDNA variants present below the limit of detection (7). To overcome this limitation, Adalsteinsson et al. pre-selected samples with 10% tumor fraction as a cutoff value using ultralow-pass whole-genome sequencing and showed that only 34% of cfDNA samples from metastatic breast- and prostate cancer patients were feasible for WES analysis (3). Technical feasibility of WES of cfDNA in previous studies has been performed on 303 samples, with a median coverage of 137x (range: 43–500x) (7). In our study, we performed WES to obtain mean 100x coverage, but sequencing coverage was highly variable, ranging from 20.1 to 211.5x coverage, in total 210 paired cfDNA samples. To achieve mean 100x coverage, re-sequencing was performed on sample that could expect increased coverage with low PCR duplicates. Finally, 152 samples were used to achieve a median 130.5x (mean = 134.5x) and 110 paired serum samples were used for the downstream analysis.

Several studies have compared between serum and plasma as use of ctDNA sources (53, 54). The cfDNA yield was higher in plasma from patients with lung cancer or colorectal carcinoma than in healthy controls. Although mutations were identified in both plasma and serum and the median molecular sequencing depth was comparable, more mutations were found in plasma than in serum and the allele frequency was higher in plasma than in serum. Those reports suggest that plasma is clearly more preferable for prospective clinical applications of liquid biopsy. But when our study had started, serum was chosen because it showed higher amount total cfDNA than plasma. Thus, the result of somatic variants calling and allelic fraction might be affected due to being diluted by DNA of non-cancerous origins.

The amount of cfDNA released by tumors is not only dependent on size, but also on turnover activity, proliferation rate, vascularization, and perfusion (51). Therefore, different tumor types of the same size can release different amounts of cfDNA. Bettegowda et al. reported that a fraction of patients with detectable ctDNA varied with tumor type (32). In this study, serum cfDNA were drawn before and after the development of drug resistance of lung and colorectal cancer patients. Although there was no significant difference between lung and colorectal cancer, mean cfDNA amount was higher in the group with larger size of residual tumor (**Supplementary Figure S1B**). It might be affected by the cfDNA from non-cancerous origin as described above. Nonetheless, the number of somatic variants was associated with ctDNA fraction and residual tumor size in colorectal cancer, but not in lung cancer (**Supplementary Figure S1A**). This could be explained by factors of ctDNA release from the tumor, so-called “ctDNA shed” (55). ctDNA shedding is related not only to tumor size and necrosis, but also the vascularity of tumor. Comprehensive histopathological features of shedding tumors in lung and colorectal cancers were not evaluated. Nevertheless, ctDNA might be released less in lung cancer than in colorectal cancer, due to the physiological conditions such as alveolar region, which is only an efficient region for gas exchange while colorectal tissue has a good network of blood vessels. Besides, multiple metastatic sites that have risen in colorectal cancers may affect more detectable somatic variants than lung cancer. Previously, our group reported that high cfDNA concentrations had significantly shorter PFS and OS than those with low cfDNA concentrations (31). In this study, patients with low ctDNA amount at resistant time point showed longer survival probability but lack statistical significance (**Supplementary Figure S1C**).

Recently, it has been reported that the transformation of EGFR-mutant lung cancer from adenocarcinoma to small-cell lung cancer at the time of acquired resistance is associated with the appearance of APOBEC mutational signatures (56). Isozaki et al. observed increased APOBEC mutational signatures in resistant tumors after TKI treatment and suggest stepwise development of mutations (56). However, no increase in APOBEC mutational signatures was also observed in metastatic sites from a patient with a shorter response to EGFR TKIs (56). In our results, lung cancer patients showed higher APOBEC signature in baseline compared with resistance to the EGFR-TKI group. These results indicate that resistant subclones of our lung cancer patients with EGFR-TKI treated group might be from independent APOBEC-driven clonal evolution during acquired resistance.

It was not surprising to see APC, TP53, KRAS, and SMAD4 as frequently mutated genes in colorectal cancer where such mutations were reported as key driver genes in progression and metastasis (57). Also, TP53 and EGFR have been identified as one of several driver mutations in NSCLC (58), and were frequently detected in our lung cancer samples, indicating the reliability of our current WES study using cfDNA. GPR155 mutation was frequently detected in acquired resistance time point in lung cancer patients only in the EGFR-TKI treated group. GPR155 encodes G protein-coupled receptor 155, and reported that mutations in this gene may be associated with autism (59, 60). Although there has been a report that GPR155 expression is suppressed in neoplasm of the thyroid, hepatocellular carcinoma, and gastric cancer, implying a tumor suppressive function for this gene, the resistant role in lung cancer, however, was not reported (61, 62). In the COSMIC, we found that GPR155 I357S mutations were estimated as pathogenic (score 0.99) and patients with this gene alteration showed poor prognosis compared those with unaltered patients. Hence, it is worthwhile to further investigate the mechanistic roles of GPR155 I357S mutation in drug resistance of lung cancer patients especially EGFR-TKI.

ADAMTS20 gene is a member of the ADAMTS family of zinc-dependent proteases. As an anti-angiogenic member of the family, ADAMTS20 was found to be downregulated in colorectal cancer (43). Mutations in the gene encoding the giant skeletal muscle protein titin (TTN) were reported that associated with several muscle disorders and were frequently detected in solid tumors (44, 63). In colorectal cancer, TTN was identified as the most frequently mutated gene within the pan-cancer cohort, and its mutation number showed the best correlation with TMB (45). Other researchers also observed that TTN, OBSCN, and ADAMTS12 genes were frequently mutated in cfDNA WES although tumor types are different as HCC (64). The association between those mutated genes and drug resistance is not clear in colorectal cancer yet. Recent reports identified that TTN mutations were associated with the largest number of resistant and sensitive drugs (65). Further study of these mutations in colorectal cancer with drug resistance could shed important light on the value of these mutations.

Several genes with high-frequency and important CNVs, namely, MYC, FGFR1, CCNE1, and CCND3 have been observed in lung and colorectal cancer samples. CCNE1 is involved in the cell cycle pathway, and its amplification has been identified in multiple cancers. Among the known driver CNVs found in lung cancer sample, the copy number of MYC and FGFR1 increased in the resistant time point. Schaub et al. described that MYC is the most frequently amplified gene among the proximal network members across all cancer types, and suggest that MYC is a distinct oncogenic driver (66). Increased FGFR1 expression is frequent across various lung cancer histologies, namely, squamous cell carcinomas and adenocarcinomas (67). These genes with CNVs in lung cancer might be potential therapeutic targets.

In conclusion, our study identified the somatic variant associated with drug resistance from lung and colorectal cancer patients using WES and provided a genetic profile. We conclude that cfDNA could be used to identify somatic variants associated with acquired resistance to treatment of lung cancer and colorectal cancer, which could guide change regimen when those biomarkers were detected in the blood.

## Data Availability Statement

The original contributions presented in the study are included in the article/**Supplementary Material**. Further inquiries can be directed to the corresponding author.

## Ethics Statement

The studies involving human participants were reviewed and approved by the Institutional Review Board of the Korea University Medical Center (ED14110). The patients/participants provided their written informed consent to participate in this study.

## Author Contributions

Study conception and design: YHK. Patient recruitment and clinical data collection: JYC, WJC, SL, SYL, and YHK. cfDNA isolation and experimental data acquisition: JWL, YSP, JSS, BK, and SBL. WES data analysis and interpretation: JC. Manuscript writing: JWL. Manuscript revision and intellectual contribution: JC and YHK. Study supervision: YHK. All authors listed have made a substantial, direct, and intellectual contribution to the work and approved it for publication.

## Funding

This research was supported by the Korea Health Technology R&D Project through the Bio & Medical Technology Development Program and Basic Science Research Program of the National Research Foundation funded by the Korean Government, Ministry of Science, ICT and Future Planning (NRF-2015M3A9D7031070, NRF-2017R1A-2B3004624).

## Conflict of Interest

The authors declare that the research was conducted in the absence of any commercial or financial relationships that could be construed as a potential conflict of interest.

## Publisher’s Note

All claims expressed in this article are solely those of the authors and do not necessarily represent those of their affiliated organizations, or those of the publisher, the editors and the reviewers. Any product that may be evaluated in this article, or claim that may be made by its manufacturer, is not guaranteed or endorsed by the publisher.

## Supplementary Material

Supplementary Figure 1 Clinical relations of somatic alterations detected in cfDNA from lung and colorectal cancer patients. **(A)** Correlations between ctDNA fraction and clinical information regarding lung cancer and colorectal cancer samples. **(B)** Total Serum cfDNA depending on tumor type and size. Sum of the longest diameter (mm) residual tumors in each type of patient were compared (small = sum of the longest two tumors < 3 cm and large = sum of the longest two tumors ≥ 3 cm). **(C)** Kaplan-Meier estimates of overall survival (OS) according to the amount of ctDNA. Samples were categorized into two subgroups by median ctDNA amount (Lung = 6.65 ng and Colon = 8.88 ng).

Supplementary Figure 2 Characteristics of potential somatic SNVs. **(A)** Comparison between the observed distribution of somatic SNVs across the 96 possible mutation types and summation of the distribution of the decomposed signature. **(B)** Distribution of microsatellite instability (MSI) percentages generated from the cfDNA WES data using msisensor2.

Supplementary Figure 3 Comparing mutation allelic fraction quantified from serum by WES and ddPCR. **(A)** Mutational profile and variant allelic fraction determined by WES and ddPCR in 14 selected serum samples. **(B)** Pearson correlation based on the mutant allelic fraction of the standard in cfDNA samples.

Supplementary Table 1 Somatic variants of 276 DDR genes specific to DNA damage repair pathways in hypermutated samples are listed in 36. Cell Reports.

Supplementary Table 2 Clinical relevance of genes with alterations. Potentially functional and known disease-related variants in the ClinVar and COSMIC databases.

Supplementary Table 3 Genes with CNVs detected in cfDNA.
