# Supplementary material for: Genetic Characteristics Associated With Drug Resistance in Lung Cancer and Colorectal Cancer Using Whole Exome Sequencing of Cell-Free DNA
Source: Front Oncol. 2022 Mar 24;12:843561. doi: 10.3389/fonc.2022.843561 (PMC8987589; doi:10.3389/fonc.2022.843561)
Supplement: Supplementary Figure 1 — Clinical relations of somatic alterations detected in cfDNA from lung and colorectal cancer patients. (A) Correlations between ctDNA fraction and clinical information regarding lung cancer and colorectal cancer samples. (B) Total Serum cfDNA depending on tumor type and size. Sum of the longest diameter (mm) residual tumors in each type of patient were compared (small = sum of the longest two tumors < 3 cm and large = sum of the longest two tumors ≥ 3 cm). (C) Kaplan-Meier estimates of overall survival (OS) according to the amount of ctDNA. Samples were categorized into two subgroups by median ctDNA amount (Lung = 6.65 ng and Colon = 8.88 ng). [file Presentation_1.pptx]

## Slide 1
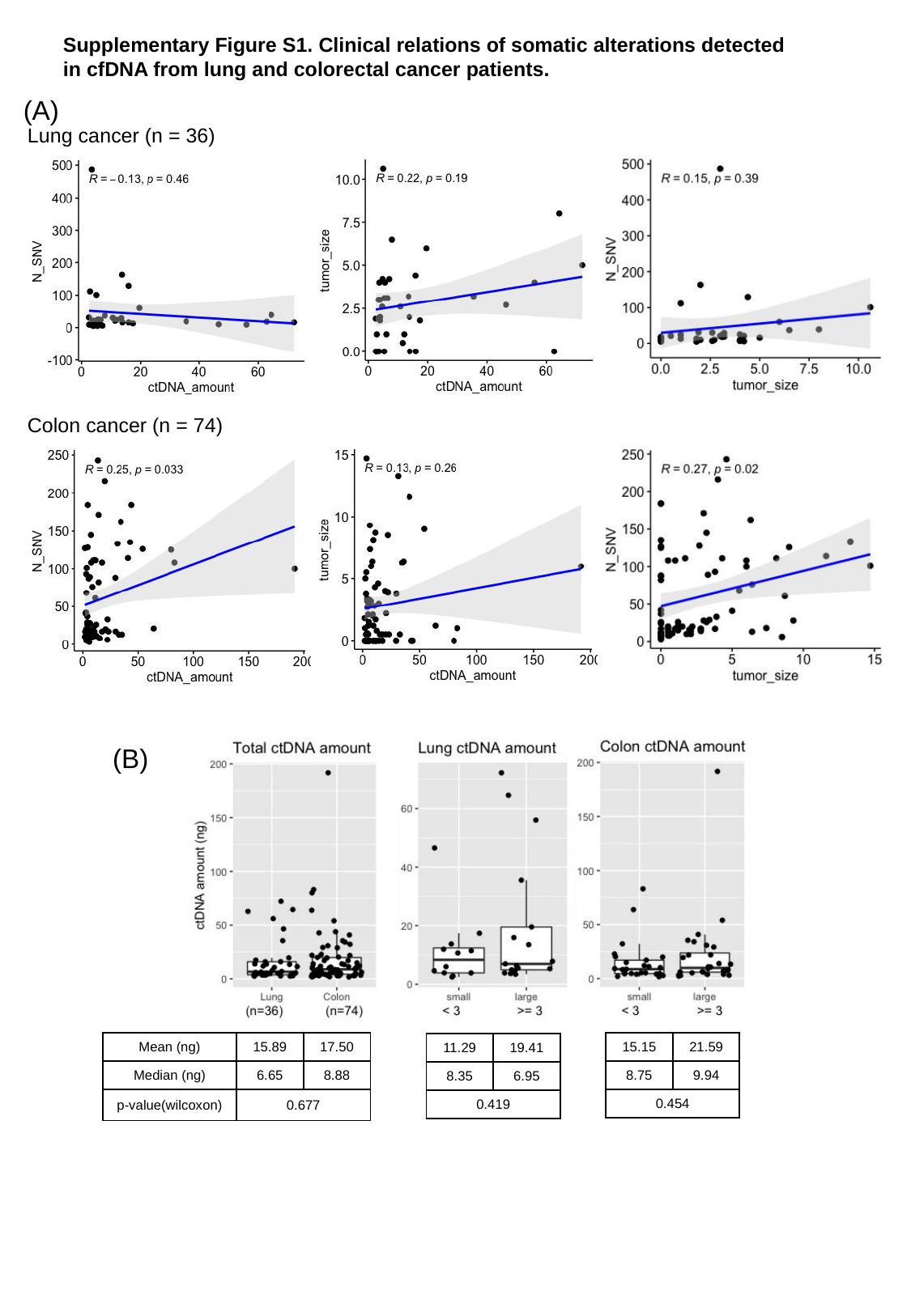

Supplementary Figure S1. Clinical relations of somatic alterations detected in cfDNA from lung and colorectal cancer patients.
(A)
Lung cancer (n = 36)
Colon cancer (n = 74)
(B)
| Mean (ng) | 15.89 | 17.50 |
| --- | --- | --- |
| Median (ng) | 6.65 | 8.88 |
| p-value(wilcoxon) | 0.677 | |
| 15.15 | 21.59 |
| --- | --- |
| 8.75 | 9.94 |
| 0.454 | |
| 11.29 | 19.41 |
| --- | --- |
| 8.35 | 6.95 |
| 0.419 | |

## Slide 2
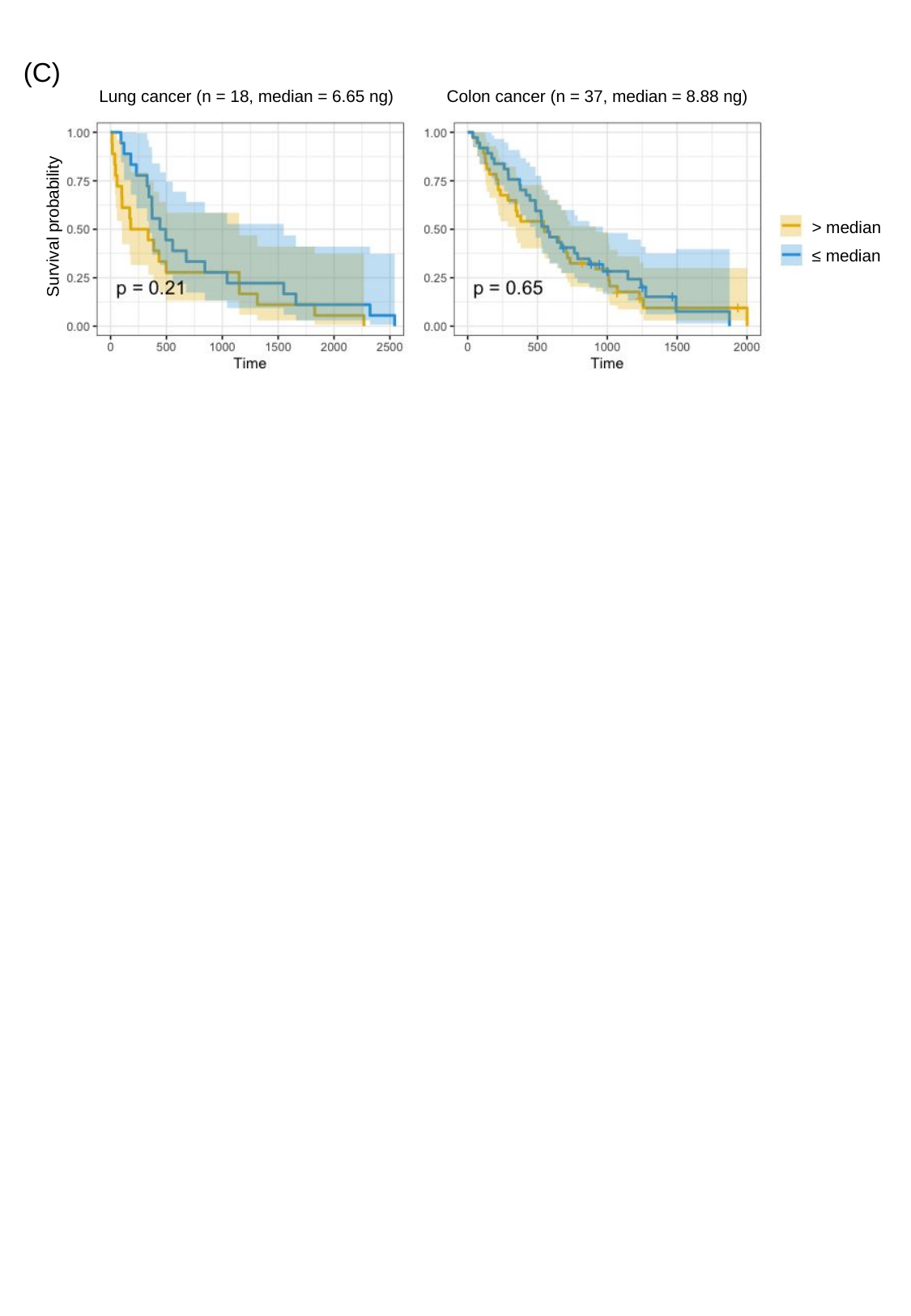

(C)
Lung cancer (n = 18, median = 6.65 ng)
Colon cancer (n = 37, median = 8.88 ng)
> median
Survival probability
≤ median

## Slide 3
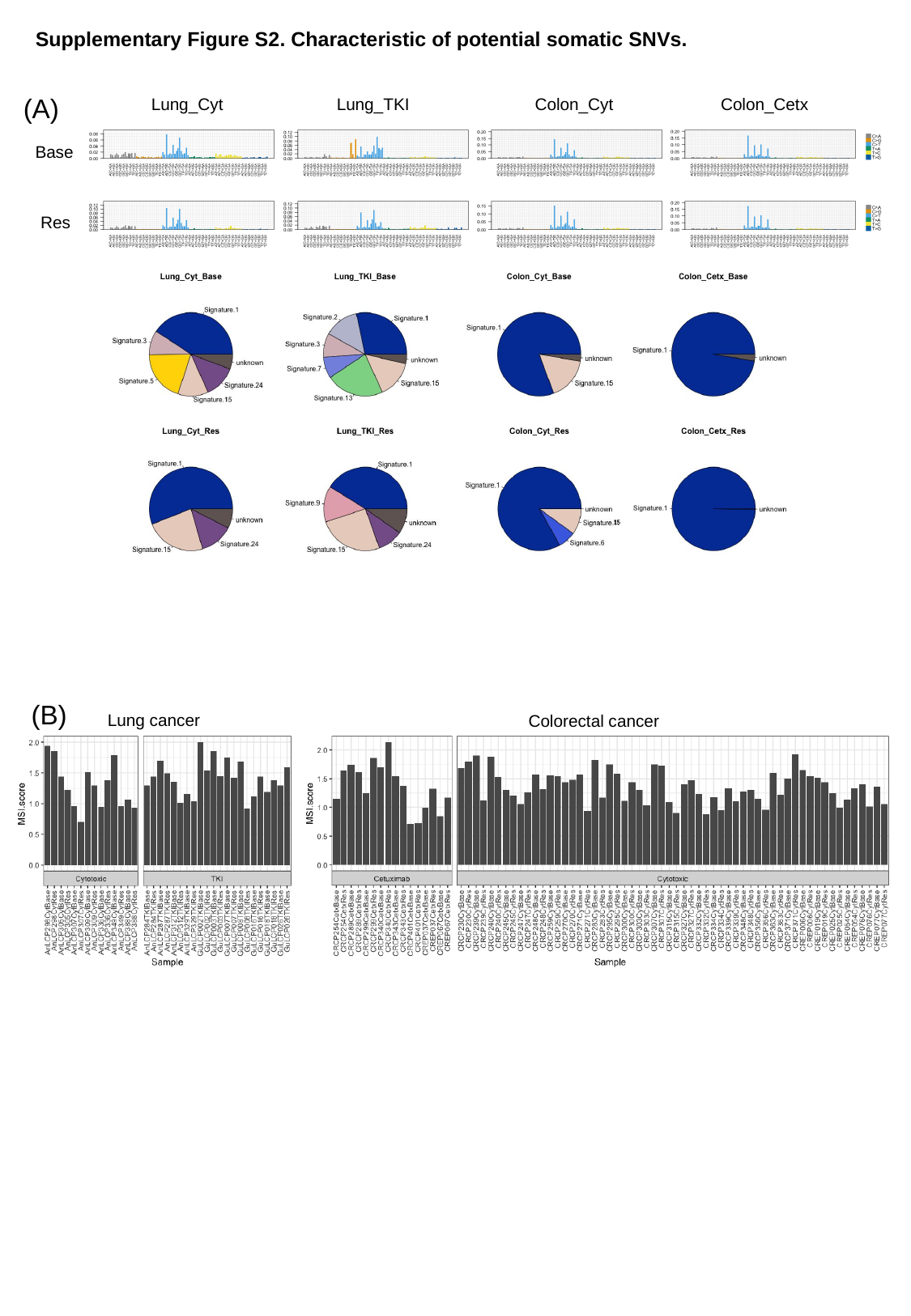

Supplementary Figure S2. Characteristic of potential somatic SNVs.
(A)
Colon_Cetx
Lung_TKI
Colon_Cyt
Lung_Cyt
Base
Res
15
(B)
Lung cancer
Colorectal cancer

## Slide 4
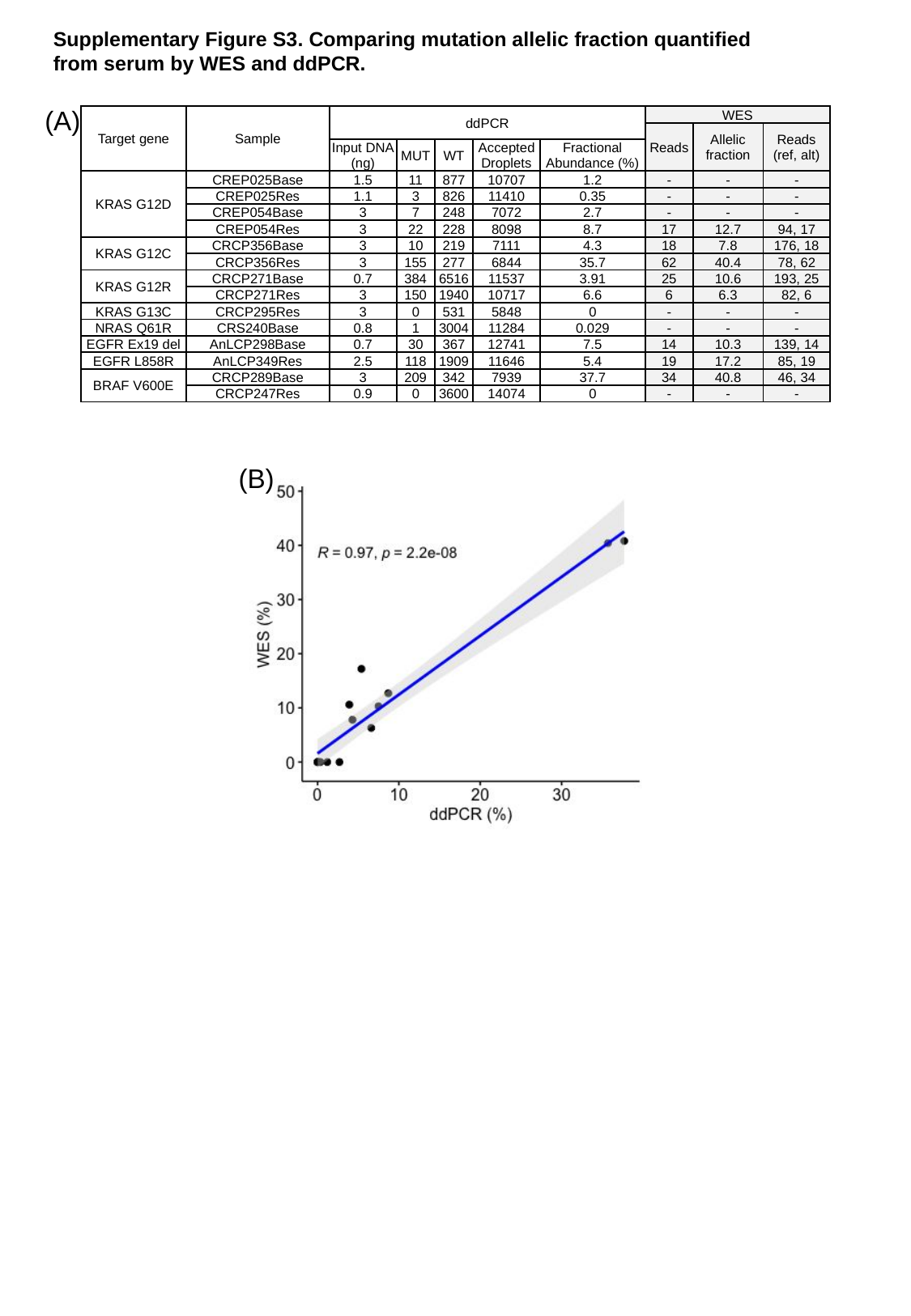

Supplementary Figure S3. Comparing mutation allelic fraction quantified from serum by WES and ddPCR.
(A)
| Target gene | Sample | ddPCR | | | | | WES | | |
| --- | --- | --- | --- | --- | --- | --- | --- | --- | --- |
| | | | | | | | Reads | Allelic fraction | Reads (ref, alt) |
| | | Input DNA (ng) | MUT | WT | Accepted Droplets | Fractional Abundance (%) | | | |
| KRAS G12D | CREP025Base | 1.5 | 11 | 877 | 10707 | 1.2 | - | - | - |
| | CREP025Res | 1.1 | 3 | 826 | 11410 | 0.35 | - | - | - |
| | CREP054Base | 3 | 7 | 248 | 7072 | 2.7 | - | - | - |
| | CREP054Res | 3 | 22 | 228 | 8098 | 8.7 | 17 | 12.7 | 94, 17 |
| KRAS G12C | CRCP356Base | 3 | 10 | 219 | 7111 | 4.3 | 18 | 7.8 | 176, 18 |
| | CRCP356Res | 3 | 155 | 277 | 6844 | 35.7 | 62 | 40.4 | 78, 62 |
| KRAS G12R | CRCP271Base | 0.7 | 384 | 6516 | 11537 | 3.91 | 25 | 10.6 | 193, 25 |
| | CRCP271Res | 3 | 150 | 1940 | 10717 | 6.6 | 6 | 6.3 | 82, 6 |
| KRAS G13C | CRCP295Res | 3 | 0 | 531 | 5848 | 0 | - | - | - |
| NRAS Q61R | CRS240Base | 0.8 | 1 | 3004 | 11284 | 0.029 | - | - | - |
| EGFR Ex19 del | AnLCP298Base | 0.7 | 30 | 367 | 12741 | 7.5 | 14 | 10.3 | 139, 14 |
| EGFR L858R | AnLCP349Res | 2.5 | 118 | 1909 | 11646 | 5.4 | 19 | 17.2 | 85, 19 |
| BRAF V600E | CRCP289Base | 3 | 209 | 342 | 7939 | 37.7 | 34 | 40.8 | 46, 34 |
| | CRCP247Res | 0.9 | 0 | 3600 | 14074 | 0 | - | - | - |
(B)
